# Psychological Impact of the COVID-19 Pandemic on Adults and Their Children in Italy

**DOI:** 10.3389/fpsyt.2021.572997

**Published:** 2021-03-12

**Authors:** Chiara Davico, Ada Ghiggia, Daniele Marcotulli, Federica Ricci, Federico Amianto, Benedetto Vitiello

**Affiliations:** ^1^Section of Child and Adolescent Neuropsychiatry, Department of Public Health and Pediatric Sciences, University of Turin, Turin, Italy; ^2^Department of Psychology, University of Turin, Turin, Italy; ^3^Department of Neuroscience, University of Turin, Turin, Italy

**Keywords:** children, COVID-19, healt h care workers, pandemic, psychological impact

## Abstract

**Aim:** The coronavirus disease 2019 (COVID-19) pandemic has abruptly changed the life of millions as travel and social contacts have been severely restricted. We assessed the psychological impact of COVID-19 on adults and children, with special attention to health care workers (HCWs).

**Methods:** A self-rated online survey, including the Impact of Event Scale-Revised (IES-R) for adults and the Children Revised Impact of Event Scale-Revised-13 items (CRIES-13) for their 8–18-year-old offspring, was conducted in Italy on March 20–26, 2020. Linear mixed-effects models were applied to the data, accounting for age, sex, education, and other demographic characteristics.

**Results:** Data were available from 2,419 adults (78.4% females, mean age 38.1 ± SD 13.1 years; 15.7% HCW) and 786 children (50.1% male, mean age 12.3 ± 3.2 years). Median (IQR) IES-R score was 30.0 (21.0–40.0), corresponding to mild psychological impact, with 33.2% reporting severe psychological impact. IES-R was lower in HCWs (29.0) than non-HCWs (31.0), but HCWs directly involved in COVID-19 care had higher scores [33.0 (26.0–43.2)] than uninvolved HCWs [28.0 (19.0–36.0)]. Median CRIES-13 score was [21.0 (11.0–32.0)], with 30.9% of the children at high risk for post-traumatic stress disorder. Parent and child scores were correlated.

**Conclusions:** Up to 30% of adult and children in the pandemic area are at high risk for post-traumatic stress disturbances. The risk is greater for HCWs directly involved in COVID-19 care and for their children.

## Introduction

On the 11th of March 2020, the WHO reported that the coronavirus disease 2019 (COVID-19) had become a pandemic, involving 114 countries and more than 118,000 cases. Italy, with 42,220 cases and 3,200 deaths as of March 20, 2020 (1) had the second highest number of COVID-19 cases worldwide, after China (2). On March 10th, in an attempt to contain the spreading of the infection, the Italian government closed all non-essential businesses and services, including also schools, universities, parks, theaters, and museums, and imposed severe limitations on the freedom to move and interact socially. In the following days, these public health dispositions were further tightened so that the entire Italian population was put on a lockdown.

Despite all efforts to contain the infection, the Italian National Health Care System was severely tested and health care workers (HCWs) overwhelmed by the demand (3). As previously happened in Wuhan, during the peak of the COVID-19 outbreak, HCWs faced a particularly stressful situation, with high risk of infection, inadequate access to protective devices, and social isolation, with consequent emergence of anxiety and depressive symptoms (4–7). These mental health problems can not only affect HCWs' attention, understanding, and decision-making ability, but also have lasting consequences for their well-being. During the 2003 outbreak of severe acute respiratory syndrome (SARS), a similar but much more limited epidemic, high levels of psychological distress and post-traumatic stress symptomatology (PTSS) were reported among HCWs (8, 9). A study conducted by Wang and colleagues 2 weeks into the China's outbreak of COVID-19 found that about half of the surveyed HCWs reported moderate to severe psychological impact, with about one-third reporting moderate to severe anxiety symptoms (10).

While data are available on the impact of the pandemic on HCWs (6, 7), little is known about possible effect on children, and in particular the children of HCW directly involved in COVID-19 care. In fact, since the beginning of the pandemic, more than 15,000 HCWs had been infected and 109 had died as of April 10, 2020 (11). Another relevant and yet unexplored issue is the worry that parents, and particularly HCWs, may have of infecting their children and of possible long-term consequences of COVID-19 (12, 13).

In this study, we evaluated the psychological impact of the COVID-19 pandemic on a sample of adults and their children, with special attention to HCWs, during the first 2 weeks of the COVID-19 outbreak in Italy, at time when the entire country was on general lockdown. We hypothesized that HCWs involved in COVID-19 care and their children would have greater indexes of psychological distress.

## Materials and Methods

### Design and Participants

We conducted a cross-sectional survey among the general public in Italy during the peak of the COVID-19 pandemic to assess adult and child psychological response through an anonymous online questionnaire. A snowball strategy was adopted. The online survey was first spread through WhatsApp among HCW colleagues and acquaintances in the North-West of Italy, encouraging them to pass it on to others, health professionals or not. Participants gave informed consent and completed the survey via an online platform (Google Forms, Google LLC, 1600 Amphitheater Parkway, Mountain View, CA 94043, USA). Participants who had children between 8 and 18 years of age were instructed to have them complete the child survey (CRIES-13). Expedited approval was obtained from the institutional ethics committee. Data were collected between 3 P.M. of the 20th March 2020 and 6 P.M. of the 26th.

### Assessments

Participants provided information about their age, gender, birthplace, residence area, education level, marital status, and any offspring between 8 and 18 years of age. Participants also were asked about place of work and whether they or their family partner were HCWs (physician or nurse) and directly involved in providing COVID-19-related care. Participants were also queried about having tested positive to the virus, or if any relative or friend had contracted COVID-19. Work exposure to COVID-19 was coded yes/no, and extent of daily exposure to COVID-19 patients was rated on Likert scale from never to always. As we were in a very early stage of the pandemic, we only inquired whether participants' close relatives had tested positive to the COVID-19 virus, as death from the disease was still a relatively rare event.

The psychological impact of COVID-19 among adults was measured on the Impact of Event Scale-Revised (IES-R) (14). The IES-R is a self-administered questionnaire that has been validated in the Italian population (15) to measure post-traumatic stress symptomatology in the past seven days. It is a 22-item questionnaire on a five-point Likert scale (0–4, with labels of “Not at all” to “Extremely”) with three subscales measuring avoidance, intrusion, and hyperarousal, and generating a total score. Total IES-R score can be considered normal (0–23) or indicative of mild (24–32), moderate (33–36), or severe (≥37) psychological impact. In our sample, the IES-R Cronbach's alpha was excellent (α = 0.91).

Children completed the Children's Revised Impact of Event Scale (CRIES-13), which is a 13-item scale adapted from the Impact of Event Scale (IES) (16, 17). It is widely used to screen children at high risk for post-traumatic stress disorder (PTSD). Items are rated on a four-point Likert scale (None = 0, Rarely = 1, Sometimes = 3, and A lot = 5), according to the frequency of recurrence of post-traumatic stress reactions during the past week, as well as in relation to a specific traumatic event noted at the top of the scale. The total score can range from 0 to 65, and is obtained from the scores of the three subscales (intrusion, avoidance, and arousal). A cut-off of 30 identifies children at risk for PTSD (16). In the study sample, CRIES-13 had a high level of internal consistency, as shown by a Cronbach's alpha of 0.86.

### Statistical Analyses

Statistical analyses were performed using the statistical programming language “R” (version 3.5.1) (18). Descriptive statistics were calculated for sociodemographic characteristics, current job activity, and risk exposure to COVID-19 (Tables 1, 2). Continuous variables were described by median and interquartile range (IQR). Categorical data were expressed as percentage. Linear mixed models (lme4 package) were used to identify variables associated with IES-R and CRIES-13 scores (19). Separate models were run for adults and children. The specified model for adults had fixed effects for HCW (yes/no) and CoV-SARS2 exposure (high/low). As HCWs were expected to have higher exposure than non-HCWs, the interaction between these two factors was taken into account by including a further fixed effect. The difference between HCWs currently employed in COVID-19 wards and those uninvolved in direct COVID-19 care was modeled with the use of a second model with fixed effect for COVID-19 ward employment (yes/no). Both models were adjusted for workplace, gender, educational attainment and age (categorized).

**Table 1:** Adults: socio-demographics and psychological impact (IES-R).

	**No. (%)**		
**Variable**	**All subjects *N* = 2,419**	**HCWs *N* = 380**	**Non-HCWs *N* = 2,039**
**Gender**			
Male	522 (21.6)	84 (22.1)	438 (21.5)
Female	1,897 (78.4)	296 (77.9)	1,601 (78.5)
**Age, y**			
18–29	802 (33.2)	84 (22.1)	718 (35.2)
30–49	1,102 (45.6)	216 (56.8)	886 (43.5)
50–69	493 (20.4)	77 (20.3)	416 (20.4)
Over 70	22 (0.8)	3 (0.8)	19 (0.9)
**Marital status**			
Single	937 (38.7)	129 (33.9)	808 (39.6)
Married/cohabitant	1,337 (55.3)	227 (59.8)	1,110 (54.4)
Divorced/separated	123 (5.1)	21 (5.5)	102 (5.0)
Widowed	22 (0.9)	3 (0.8)	19 (1.0)
**Education level (ISCED level)**			
Pre-primary/primary education (0/1)	7 (0.3)	0 (0)	6 (0.3)
Lower secondary education (2)	149 (6.2)	1 (0.3)	148 (7.3)
Upper/post-secondary education (3/4)	811 (33.5)	23 (6.2)	788 (38.6)
First tertiary education (5)	1,048 (43.3)	163 (42.9)	885 (43.4)
Second tertiary education (6)	404 (16.7)	192 (50.6)	212 (10.4)
**Place of working activity in Italy**			
North	2,086 (86.2)	314 (82.6)	1,784 (85.7)
Central	220 (9.1)	47 (12.4)	167 (8.2)
South	113 (4.7)	19 (5.0)	124 (6.1)
**Your partner is an HCW:**			
Yes	–	109 (28.7)	–
No	–	191 (50.2)	–
Single	–	80 (21.1)	–
**Your partner is daily exposed to Covid-19:**			
Yes	–	48 (12.6)	–
No	–	252 (66.3)	–
Single	–	80 (21.1)	–
**Someone close to you is Covid-19+?**			
Yes	632 (26.1)	160 (42.1)	472 (23.1)
No	1,787 (73.9)	220 (57.9)	1,567 (76.9)
**How often are you exposed to Covid-19?**			
Never	415 (17.2)	15 (3.9)	400 (19.6)
Sometimes	1,604 (66.3)	193 (50.8)	1,411 (69.2)
Often	361 (14.9)	154 (40.6)	207 (10.2)
Always	39 (1.6)	18 (4.7)	21 (1.0)
**Number of sons aged 8–18**			
1 son	334 (13.8)	50 (13.2)	284 (13.9)
2 sons	183 (7.6)	28 (7.4)	155 (7.6)
More than 2	43 (1.8)	7 (1.8)	36 (1.8)
No sons aged 8–18	1,859 (76.8)	295 (77.6)	1,564 (76.7)
**IES-R, median (IQR)**			
Total score	30.0 (21.0–40.0)	29.0 (21.0–40.0)	31.0 (21.0–40.0)
Intrusion	11.0 (7.0–15.0)	11.0 (7.0–16.0)	11.0 (7.0–15.0)
Avoidance	11.0 (8.0–15.0)	10.0 (7.0–14.0)	12.0 (8.0–15.0)
Hyperarousal	8.0 (5.0–12.0)	8.0 (5.0–11.0)	8.0 (5.0–12.0)

*HCW, health care worker; IES-R, 22-item Impact of Event Scale–Revised; IQR, interquartile range; ISCED, International Standard Classification of Education*.

**Table 2:** Children: demographics and psychological impact (CRIES-13).

**Variable**	**All subjects** ***N* = 786**	**HCW parent** ***N* = 120**	**Non-HCW parent** ***N* = 666**
**Gender**			
Male	394 (50.1)	56 (46.7)	338 (50.8)
Female	392 (49.9)	64 (53.3)	328 (49.2)
**Age, y**			
8–10	288 (36.6)	45 (37.5)	243 (36.5)
11–13	203 (25.8)	34 (28.3)	169 (25.4)
14–16	187 (23.8)	24 (20)	163 (24.5)
17–18	108 (13.8)	17 (14.2)	91 (13.6)
**CRIES-13, median (IQR)**			
Total score	21.0 (11.0–32.0)	21.0 (9.0–31.7)	21.5 (12.0–32.2)
Intrusion	6.0 (2.0–10.0)	6.0 (2.0–10.0)	6.0 (2.0–10.0)
Avoidance	6.5 (1.0–12.0)	5.0 (0.0–11.0)	7.0 (2.0–12.0)
Arousal	8.0 (3.0–13.0)	7.0 (3.0–14.0)	8.0 (4.0–13.0)

*HCW, health care worker; CRIES-13, Children's Revised Impact of Event Scale 13-item; IQR, interquartile range*.

As parental stress could influence children, offspring stress expression models included IES-R as a fixed effect (20). Child age and having or not a HCW parent were the other fixed effect factors. To verify whether there was an association between siblings' psychological impact, we added sibling's CRIES-13 for fixed effect in the analysis of data from families with more the one child aged 8–18 years. To this end, we alternatively used the CRIES-13 score of one sibling (sibling 1) as the outcome measure and the score of other (sibling 2) as a predictor. The difference between children of HCWs currently employed in COVID-19 wards and children of other HCWs was modeled with the use of a third model with fixed effect for parents' COVID-19 ward employment (yes/no). Random effects in all the offspring models were: parent education, parent's workplace, gender, number of siblings, and parent's COVID-19 exposure intensity. Group differences were assessed with Mann Whitney *U*-test. Statistical significance was set at *p* < 0.05. To verify whether violation of the normality of residuals assumption and outliers affected the linear mixed model analyses, robustified versions of the same linear mixed models were also conducted (data not shown) (21).

## Results

### Socio-Demographics Characteristics

We received responses from 2,438 adults, of whom 19 did not give consent to the use of the data (participation rate: 99.2%). Participants were 2,419 adults (mean age 38.1 ± 13.1 year; 78.4% females) from all parts of Italy (North: *n* = 2,086, 86.2%; Central: *n* = 220, 9.1%; South: *n* = 113, 4.7%). Most participants were married or cohabitant (1,337, 55.3%), 937 (38.7%) were single, 123 (5.1%) divorced or separated, and 22 (0.9%) widowed (Table 1). Almost half of the sample (49%) had children. Of the adult participants, 380 (15.7%) were HCWs, of whom 294 (77.4%) physicians and 86 (22.6%) nurses. Of the HCWs, 122 (32.1%) were currently employed in COVID-19 wards. Only 27 subjects (1.1%) had tested positive to Covid-19. Data were collected on 786 children (394 or 50.1% males), with mean age 12.3 ± 3.2 years. Demographics are reported in Table 2.

### Psychological Impact of COVID-19

**Figure 1:**
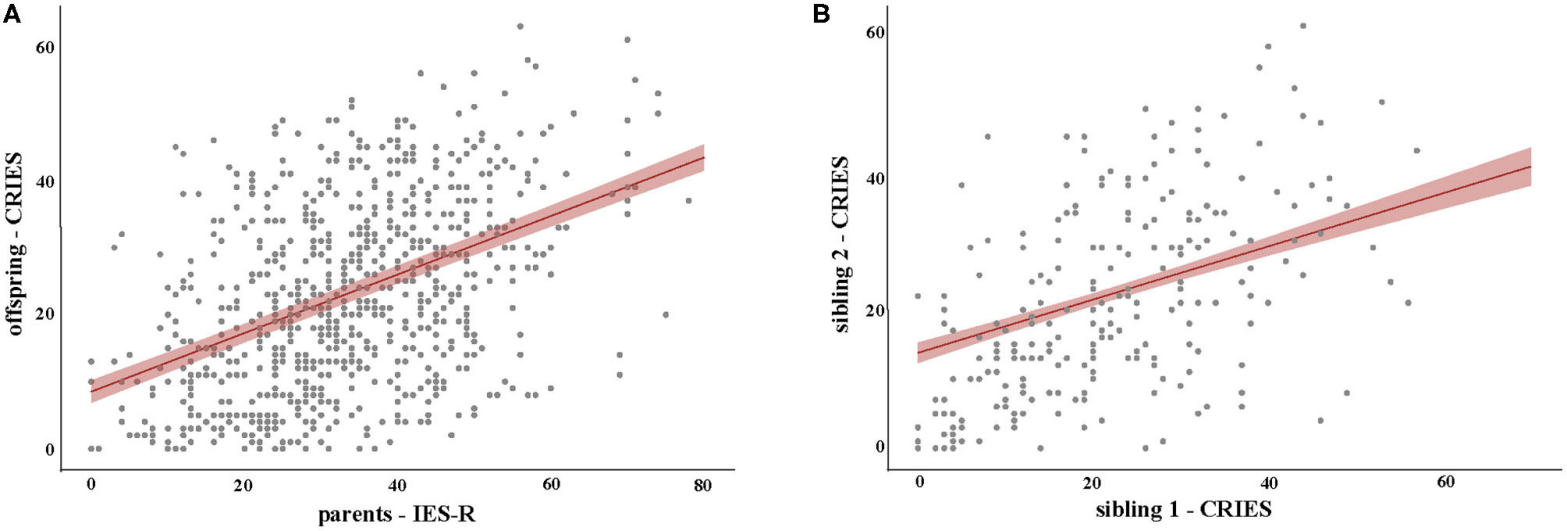
Regression lines for the IES-R **(A)** and CRIES-13 **(B)** predictors. Shadowing indicates standard errors.

In adults, the IES-R total score median (IQR) was 30.0 (21.0–40.0), corresponding to mild psychological impact (Table 1; Figure 1). For 30.4%, the, IES-R score was in the normal range (0–23). One third (33.2%) had a score consistent with severe psychological impact (i.e., IES-R ≥ 37), with no significant difference between HCW (29.7%) and non-HCW participants (33.8%). However, HCWs involved in direct COVID-19 care had higher median IES-R scores [33.0 (26.0–43.2)] than uninvolved HCWs [28.0 (19.0–36.0)]. Having a relative who had tested positive to SARS-CoV-2 was not associated with a higher IES-R score.

In children, the CRIES-13 total score median (IQR) was [21.0 (11.0–32.0)], i.e., below the cut-off of 30 for being at risk for PTSD. For 30.9% the CRIES-13 score was 30 or greater (Table 2). No significant differences were found between children of HCW parents [21.0 (9.0–31.7)] and those of non-HCWs [21.5 (12.0–32.2)] on the total CRIES-13 score. Furthermore, there was no significant difference between children of HCW parents who were directly involved in COVID-19 care and those of HCW parents who did not have such an involvement (**Table 5**).

### Factors Associated With Psychological Distress

In adults, being female was strongly associated with higher IES-R scores (*p* < 0.001, Mann-Whitney *U*-test) (Table 3). Thus, data were also analyzed by sex. Overall, being a HCW was associated with lower IES-R total scores [estimated mean difference −2.48 (−4.39 to −0.57)], as shown in Table 3 (Model 1). However, HCW employed in COVID-19 wards reported more distress than other HCWs [estimated mean difference 5.71 (−2.92 to 8.50), Model 2, Table 3].

**Table 3:** Factors associated with psychological impact in adults.

	**Outcome—psychological impact (IES-R)**			
	**Model 1**		**Model 2**	
**Predictor**	**Estimate**	**95% CI**	**Estimate**	**95% CI**
**Being a HCW**				
Both males and females	−2.48^*^	−4.39, −0.57	–	–
Males only	−1.64	−5.38, 2.10	–	–
Females only	−2.66^*^	−4.86, −0.46	–	–
**High exposure to Covid-19**				
Both males and females	4.95^***^	3.14, 6.76	–	–
Males only	−1.07	−5.07, 2.93	–	–
Females only	6.34^***^	4.32, 2.52	–	–
**Working on Covid-19 ward**				
Both males and females	–	–	5.71^***^	2.92. 8.50
Males only	–	–	9.97^**^	3.68, 16.26
Females only	–	–	4.70^**^	1.60, 7.80

* *p < 0.05*;

** *p < 0.01*;

*** *p < 0.001*.

In males, age or being a HCW was not associated with the reported level of distress (Table 3). But male HCWs who were employed in COVID-19 wards reported significantly higher distress than other male HCWs (Model 2, Table 3).

In females, all previously identified factors (i.e., HCW, COVID-19 exposure, and employed in COVID-19 wards) were associated with IES-R scores (Table 3).

In children, CRIES-13 scores were related to their parents' IES-R scores (see Table 4 and Supplementary Figure 1). Importantly this finding held true for both single children and for siblings (Table 4 and Supplementary Material). Additionally, siblings' CRIES-13 are correlated, suggesting a possible “family effect” for distress. In agreement with previous findings (22) and similarly to adult results, girls expressed higher distress level (*p* < 0.001, Mann-Whitney *U*-test). No other factors were significantly associated with CRIES-13 scores in our model (Tables 4, 5 and Supplementary Material).

**Table 4:** Factors associated with psychological impact in children.

	**Outcome—psychological impact (CRIES-13)**			
	**All subjects**		**Sibling−1**	
**Predictor**	**Estimate**	**95% CI**	**Estimate**	**95% CI**
Parental psychological impact (IES-R)	0.44^***^	0.31, 0.50	0.30^***^	0.19, 0.40
Age	−0.22	−0.49, 0.04	0.08	−0.38, 0.53
Have a HCW parent	−1.73	−3.95, 0.48	−0.35	−3.95, 3.24
Sibling−2 psychological impact (CRIES-13)	–	–	0.38^***^	0.28, 0.49

*** *p < 0.001*.

**Table 5:** Factors associated with psychological impact in HCWs' children.

	**Outcome—psychological impact (CRIES-13)**	
	**All subjects**	
**Predictor**	**Estimate**	**95% CI**
Parental psychological impact (IES-R)	0.36^***^	0.16, 0.57
Age	−0.19	−0.84, 0.47
Have a Covid-19 involved parent	1.18	−9.45, 11.80
Parental IES-R × Covid-19 involved parent	0.07	−0.22, 0.35

*** *p < 0.001*.

## Discussion

This study investigated the psychological impact of the COVID-19 epidemics on adults and children at the time of the highest daily increase in infections in Italy (11). By using a large, nationwide, self-selected sample and validated measures of psychological impact from traumatic situations, we found that about one third of the participants reported moderate-to-severe psychological distress. HCWs were not, as a group, at higher risk for psychological distress than non-HCWs, but those HCWs directly involved in providing COVID-19 care had significantly higher indexes of distress.

Children's ratings were correlated to those of their parents, and about 30% of them had indexes indicative of higher risk for post-traumatic distress. A correlation between parent and child ratings is expected, reflecting a commonality of contextual factors related to COVID-19 and a similarity in temperamental traits and emotional communication capacity that are likely to be both genetically and environmentally influenced (23). Consistent with the psychiatric literature on mood and anxiety disorders and other reports on post-traumatic stress, females reported greater psychological distress than males, in both the whole sample and the HCW subgroup (4).

The IES-R scores in our sample are consistent with those recently reported in studies of the general population (IES-R mean 32.98) and HCWs (IES-R median 21.0) in the Wuhan area in China (4, 10). Our study expands on previous reports by examining HCWs within a sample of the general population and by assessing the impact of COVID-19 on children in relation to their parents.

The results suggest that HCWs experienced, in general, less psychological distress than non-HCWs, but HCWs currently working on COVID-19 wards reported more distress, with IES-R scores indicating high risk for experiencing psychological breakdown and developing PTSD. Being directly involved in COVID-19-related healthcare was in fact the only predictor of higher distress in both males and females. Several reasons could explain these findings. On one hand, greater familiarity with health issues in general and a deeper understanding of the infection mechanisms could have helped HCWs control anxiety and reduce distress. Even during the pandemic social lockdown, HCWs were allowed to leave home and continue working, and were less restricted in social contacts than the general population, thus limiting possible feelings of boredom, frustration, and uselessness brought by the lockdown. Additionally, while many people suffered from job insecurity and faced economic uncertainty, HCWs had greater job security during the pandemic.

On the other hand, HCWs who were directly involved in COVID-19 care were more exposed to the risk of contagion and might have faced emotional pain and stress at work. During the SARS outbreak in 2003, 17.3% of the HCWs reported mental symptoms, which persisted in 15.4% at 1-year follow-up (24). In another study during the SARS outbreak in Singapore, the rate of HCWs reporting psychiatric symptoms was 17.7%, using a cut off of 26 on the IES (9). These rates are lower than in our study, possibly reflecting the extraordinary morbidity and global reach of the COVID-19 pandemic in Italy, as well as the influence of cultural factors on the perception and reporting of emotional distress.

Relatively little has been known about the psychological distress of children exposed to the pandemic. Concern has been raised that children might be particularly sensitive to the psychological effects of COVID-19 (10, 25–27). Fear of infection and home confinement could be particularly stressful for young people. Children and adolescents may be more vulnerable also because of home confinement, school closure, lack of in-person contact with classmates, friends, romantic partners, and teachers, and limitation in personal space at home (28). In this context, the role of parents becomes especially important for attenuating the psychological detrimental effects of confinement. From the child development literature, we know that children rely on trusted adults for protection and as a reference for assessing danger and attributing meaning to events (20, 29–31). Thus, it can be especially frightening for a child to perceive that the parent is distressed and unable to prevent a traumatizing event from happening. The correlation between parent (IES-R) and child (CRIES-13) psychological distress underscores the strong link existing between parent-child mental health and brings attention to the critical role of the parent in buffering the distressing effects of the pandemic and its consequences upon their children. Unlike other reports of young age being a risk factor for post-traumatic reactions (32–34), we did not find age to be a significant moderator of psychological distress in children, possibly because the sample did not include very young children.

### Limitations

This study has several limitations that must be considered in interpreting the data. First, even though the survey was widely disseminated nationwide, the sample was self-selected and not representative of the Italian population. This is evident, for example, by the 3:1 female/male ratio. This higher proportion of females is, however, comparable to previous studies on the pandemic (4, 10), thus indicating that females are more prone to complete this type of surveys. Second, the data rely on just one self-rating instrument, the IES-R for adults or the CRIES-13 for children, without other measures of current or past psychopathology. Indeed, this is a cross-sectional study and future time points will be needed to understand the psychological impact of the pandemics. Another limitation of the present study is that the IES-R had not been structured for ongoing stressful events, such as pandemics, and is not a diagnostic tool for PTSD. However, to the best of our knowledge, no more specific assessment tools have been so far validated for such events. Finally, the online survey could not control for possible heterogeneity in the way parents had their children complete the questionnaire.

### Conclusion

This study informs on the psychological impact of the COVID-19 pandemic on adults and children in Italy, with special attention to the greater risk for psychological distress among those HCWs directly involved in COVID-19 clinical care and their children. About one-third of the surveyed children reported significant distress. The close link between parent- and child-reported distress suggests that interventions aimed at preventing and managing COVID-19 related anxiety in children should take into account parental distress. Successful management of distress in parents may positively reflect on their children's mental health.

## Data Availability Statement

The raw data supporting the conclusions of this article will be made available by the authors, without undue reservation.

## Ethics Statement

The studies involving human participants were reviewed and approved by Ethical Committee of the AOU Città della Salute e della Scienza di Torino. Written informed consent to participate in this study was provided by the participants' legal guardian/next of kin.

## Author Contributions

CD and AG conceived and designed the study protocol with input from DM and BV. AG carried out literature searches. DM designed and carried out the statistical analysis. CD, AG, DM, and BV interpreted the data and drafted the manuscript. FR and FA supervised the writing of the manuscript. All authors critically reviewed and contribute to the final version of the paper.

## Conflict of Interest

In last 2 years, BV has received consultant fees or honoraria from Medice, Lundbeck, and Angelini Pharmaceuticals, and from law firms Goodwin & Procter and Haynes & Boone. The remaining authors declare that the research was conducted in the absence of any commercial or financial relationships that could be construed as a potential conflict of interest.
