# Supplementary material for: Psychological Impact of the COVID-19 Pandemic on Adults and Their Children in Italy
Source: Front Psychiatry. 2021 Mar 12;12:572997. doi: 10.3389/fpsyt.2021.572997 (PMC7994767; doi:10.3389/fpsyt.2021.572997)
Supplement: Supplementary file 1 [file Data_Sheet_1.docx]

**Table of Contents**

**Results for the linear mixed models (not shown in main text)**

**Table 1** Factors associated with psychological impact in single children **2**

**Table 2** Factors associated with psychological impact in sibling 2 **2**

**Random effects of the linear mixed models.** Fixed effects are presented in main text and supplementary table 1 and 2 **3**

**Figure 1.** Distribution of residuals for the main models for adults and children **5**

**Results for the robust linear mixed models**

**Table 3** Factors associated with psychological impact in adults **5**

**Table 4** Factors associated with psychological impact in men **5**

**Table 5** Factors associated with psychological impact in women **5**

**Table 6** Factors associated with psychological impact in HCWs **5**

**Table 7** Factors associated with psychological impact in children **5**

**Table 8** Factors associated with psychological impact in sibling 1  **6**

**Table 9** Factors associated with psychological impact in sibling 2 **6**

**Table 10** Factors associated with psychological impact in single children  **6**

**Table 11** Factors associated with psychological impact in HCWs’ children **6**

**References 7**

**Results for the linear mixed models.**

**Table 1.**

| **Factors associated with psychological impact in single children** | | | |
| --- | --- | --- | --- |
|  | Outcome - Psychological Impact (CRIES-13) | | |
| **Predictors** | **Estimates** | **95% CI** | **p** |
| Parental psychological impact (IES-R) | 6·27 | 4·95 – 7·59 | <0·001 |
| Age | -1·39 | -2·72 – -0·07 | 0·039 |
| Have a HCW parent | -2·60 | -5·96 – 0·76 | 0·129 |

**Table 2.**

| **Factors associated with psychological impact in sibling 2** | | | |
| --- | --- | --- | --- |
|  | Outcome - Psychological Impact (CRIES-13) | | |
| **Predictors** | **Estimates** | **95% CI** | **p** |
| Parental psychological impact (IES-R) | 0·20 | 0·08 – 0·33 | 0·001 |
| Age | 0·27 | -0·24 – 0·78 | 0·291 |
| Sibling’s psychological impact (CRIES-13) | 0·48 | 0·35 – 0·61 | <0·001 |
| Have a HCW parent | -0·83 | -4·93 – 3·26 | 0·690 |

**Random effects**

| **Factors associated with psychological impact in adults (see table 3)** | |
| --- | --- |
| **Random Effect** | **Variance (Std. Dev.)** |
| Educational qualification | 0.12 (0.35) |
| Workplace | 0.001 (0.03) |
| Age | 0.26 (0.50) |
| Parent | 0.38 (0.62) |
| Gender | 18.46 (4.29) |

| **Factors associated with psychological impact in men (see table 3)** | |
| --- | --- |
| **Random Effect** | **Variance (Std. Dev.)** |
| Educational qualification | 0 |
| Workplace | 0 |
| Age | 0.64 (0.80) |
| Parent | 0 |

| **Factors associated with psychological impact in women (see table 3)** | |
| --- | --- |
| **Random Effect** | **Variance (Std. Dev.)** |
| Educational qualification | 0.27 (0.51) |
| Workplace | 0 |
| Age | 0.07 (0.26) |
| Parent | 0.22 (0.46) |

| **Factors associated with psychological impact in HCW (see table 4)** | |
| --- | --- |
| **Random Effect** | **Variance (Std. Dev.)** |
| Educational qualification | 2.76 (1.66) |
| Workplace | 2.59 (1.61) |
| Age | 1.18 (1.09) |
| Parent | 0 |
| Gender | 15.3 (3.91) |

| **Factors associated with psychological impact in men HCW (see table 4)** | |
| --- | --- |
| **Random Effect** | **Variance (Std. Dev.)** |
| Educational qualification | 0 |
| Workplace | 0 |
| Age | 0 |
| Parent | 0 |

| **Factors associated with psychological impact in women HCW (see table 4)** | |
| --- | --- |
| **Random Effect** | **Variance (Std. Dev.)** |
| Educational qualification | 0 |
| Workplace | 0 |
| Age | 0.65 (0.80) |
| Parent | 0 |

| **Factors associated with psychological impact in children (Table 5)** | |
| --- | --- |
| **Random Effect** | **Variance (Std. Dev.)** |
| Educational qualification | 0.12 (0.35) |
| Workplace | 0.001 (0.03) |
| Parents exposure | 0.26 (0.50) |
| Gender | 2.04 (1.43) |

| **Factors associated with psychological impact in single children (Suppl. table 1)** | |
| --- | --- |
| **Random Effect** | **Variance (Std. Dev.)** |
| Educational qualification | 0·0001 (0.01) |
| Workplace | 6.84 (2.61) |
| Parents exposure | 0 |
| Gender | 2.57 (1.60) |

| **Factors associated with psychological impact in sibling -1 (see table 5)** | |
| --- | --- |
| **Random Effect** | **Variance (Std. Dev.)** |
| Educational qualification | 0.48 (0.70) |
| Workplace | 0 |
| Parents exposure | 0 |
| Gender | 0.28 (0.53) |

| **Factors associated with psychological impact in sibling -2**  **(Suppl. table 2)** | |
| --- | --- |
| **Random Effect** | **Variance (Std. Dev.)** |
| Educational qualification | 1.79 (1.34) |
| Workplace | 0·0002 (0.01) |
| Parents exposure | 0·0001 (0.01) |
| Gender | 0 |

| **Factors associated with psychological impact in children of HCW parents (table 6)** | |
| --- | --- |
| **Random Effect** | **Variance (Std. Dev.)** |
| Educational qualification | 0 |
| Workplace | 0 |
| Parents exposure | 0 |
| Gender | 1.93 (1.39) |

**Distribution of residuals for adults and offspring main models**

**Figure 1.**

**
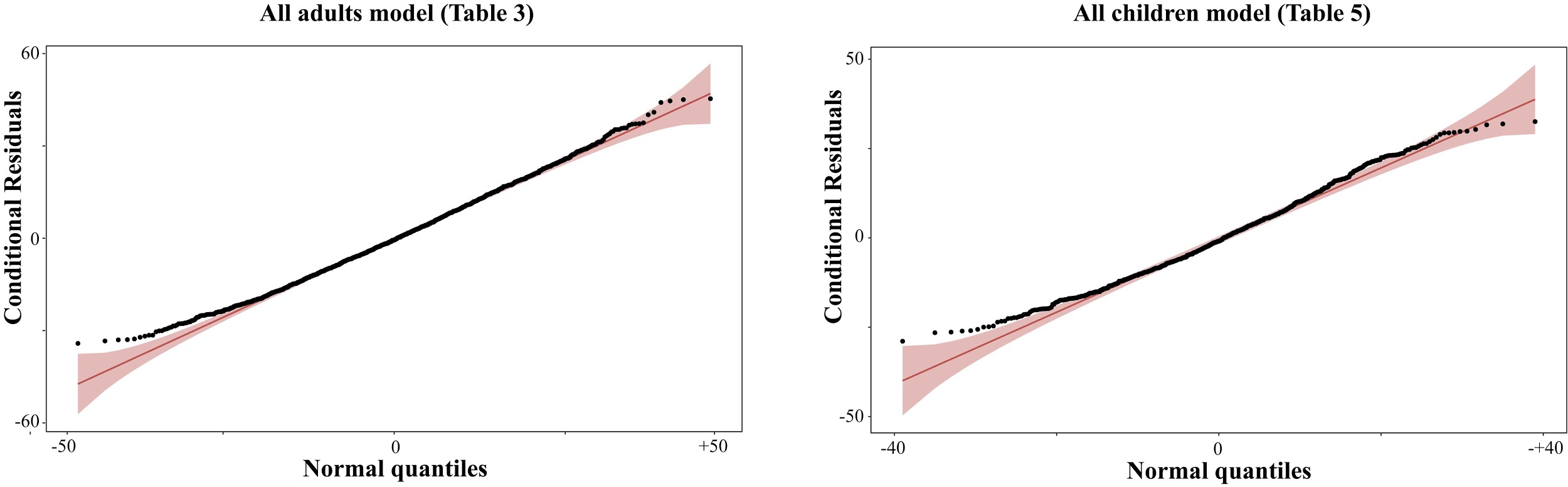
**

**Results of the robust linear mixed model (Koller, 2016)**

**Table 3.**

| **Factors associated with psychological impact in adults** | | |
| --- | --- | --- |
|  | Outcome - Psychological Impact (IES-R) | |
| **Predictors** | **Estimates** | **95% CI** |
| Being a HCW | -2·76 | -4·75 -0·77 |
| High exposure to Covid19 | 5·11 | 3·19 – 7·02 |
| Being a HCW * high exposure rate | -0·18 | -3·58 – 3·21 |

**Table 4.**

| **Factors associated with psychological impact in men** | | |
| --- | --- | --- |
|  | Outcome - Psychological Impact (IES-R) | |
| **Predictors** | **Estimates** | **95% CI** |
| Being a HCW | -2·12 | -6·07 – 1·82 |
| High exposure to Covid19 | -0·99 | -5·20 – 3·22 |
| Being a HCW * high exposure rate | 4·36 | -2·76 – 11·48 |

**Table 5.**

| **Factors associated with psychological impact in women** | | |
| --- | --- | --- |
|  | Outcome - Psychological Impact (IES-R) | |
| **Predictors** | **Estimates** | **95% CI** |
| Being a HCW | -2·91 | -5·21 – -0·61 |
| High exposure to Covid19 | 6·62 | 4·48 – 8·76 |
| Being a HCW * high exposure rate | -1·21 | -5·06 – 2·65 |

**Table 6.**

| **Factors associated with psychological impact in HCW** | | | |
| --- | --- | --- | --- |
|  | | Outcome - Psychological Impact (IES-R) | |
| **Predictors** | **Estimates** | | **95% CI** |
| Both males and females | 5·81 | | 2·85 – 8·78 |
| Males only | 11·12 | | 4·26 – 17·97 |
| Females only | 4·88 | | 1·59 – 8·17 |

**Table 7.**

| **Factors associated with psychological impact in children** | | |
| --- | --- | --- |
|  | Outcome - Psychological Impact (CRIES-13) | |
| **Predictors** | **Estimates** | **95% CI** |
| Parental psychological impact (IES-R) | 0·46 | 0·40 – 0·52 |
| Age | -0·22 | -0·51 – 0·06 |
| Have a HCW parent | -1·96 | -4·32 – 0·41 |

**Table 8.**

| **Factors associated with psychological impact in sibling 1** | | |
| --- | --- | --- |
|  | Outcome - Psychological Impact (CRIES-13) | |
| **Predictors** | **Estimates** | **95% CI** |
| Parental psychological impact (IES-R) | 0·29 | 0·19 – 0·39 |
| Age | 0·03 | -0·41– 0·47 |
| Sibling 2 psychological impact (CRIES-13) | 0·41 | 0·31 – 0·51 |
| Have a HCW parent | -0·53 | -4·06 – 3·00 |

**Table 9.**

| **Factors associated with psychological impact in sibling 2** | | |
| --- | --- | --- |
|  | Outcome - Psychological Impact (CRIES-13) | |
| **Predictors** | **Estimates** | **95% CI** |
| Parental psychological impact (IES-R) | 0·20 | 0·08 – 0·33 |
| Age | 0·35 | -0·17 – 0·87 |
| Sibling 1 psychological impact (CRIES-13) | 0·52 | 0·38 – 0·65 |
| Have a HCW parent | -0·15 | -4·45 – 4·14 |

**Table 10.**

| **Factors associated with psychological impact in single children** | | |
| --- | --- | --- |
|  | Outcome - Psychological Impact (CRIES-13) | |
| **Predictors** | **Estimates** | **95% CI** |
| Parental psychological impact (IES-R) | 6·45 | 5·02 – 7·88 |
| Age | -1·41 | -2·86 – 0·03 |
| Have a HCW parent | -2·84 | -6·50 – 0·82 |

**Table 11.**

| **Factors associated with psychological impact in offspring of HCW parents** | | |
| --- | --- | --- |
|  | Outcome - Psychological Impact (CRIES-13) | |
| **Predictors** | **Estimates** | **95% CI** |
| Parental psychological impact (IES-R) | 0·36 | 0·14 – 0·58 |
| Age | -0·12 | -0·83 – 0·59 |
| COVID-19 ward involved parent | 0·17 | -11·31 – 11·65 |
| COVID-19 ward involved parent * Parental psychological impact (IES-R) | 0·09 | -0·22-0:40 |

**References:**

Manuel Koller (2016). robustlmm: An R Package for Robust Estimation of Linear Mixed-Effects Models. Journal of Statistical Software, 75(6), 1-24. doi:10.18637/jss.v075.i06
